# Hypoxia-Induced miR-210 Promotes Endothelial Cell Permeability and Angiogenesis via Exosomes in Pancreatic Ductal Adenocarcinoma

**DOI:** 10.1155/2022/7752277

**Published:** 2022-11-25

**Authors:** Guo Wu, Xiaojie Ding, Gang Quan, Jianwei Xiong, Qiang Li, Zhonghu Li, Yaqin Wang

**Affiliations:** ^1^Department of Hepatobiliary Surgery, The Affiliated Hospital of North Sichuan Medical College, Institute of Hepatobiliary-Pancreatic-Intestinal of North Sichuan Medical College, Nanchong, China; ^2^Department of Dermatology, The Affiliated Hospital of North Sichuan Medical College, Nanchong, China; ^3^Department General Surgery, Central Theater Command General Hospital of PLA, Wuhan, China; ^4^Department of Pathology, The Affiliated Hospital of North Sichuan Medical College, Nanchong, China

## Abstract

**Background:**

Exosomes have been proven to play important diagnostic, regulatory, or communication roles in tumorigenesis, tumor progression, or metastasis; in recent studies, lots of molecules, including miRNAs, were found to be aberrantly expressed in tumor exosomes and were correlated with tumor development. However, studies about the expression, relationship, or control mechanisms of miRNAs in exosomes in pancreatic ductal adenocarcinoma (PDAC) are scarce and urgently needed. The aim of this article was to identify and investigate abnormally expressed miRNAs in PDAC exosomes *in vivo* and *in vitro*.

**Methods:**

Microarray studies were used to detect aberrantly expressed miRNAs in PDAC exosomes, and miR-210 expression in cells or exosomes was further analyzed by qRT-PCR. Bioinformatics analyses, dual-luciferase assays, WB, and other assays were utilized to explore the miRNA molecular mechanisms. The living cell coculture model and immunofluorescence analysis were employed to image the communication between PDAC cells and endothelial cells. Other biological experiments in the study include a real-time intravital imaging system, EdU, transwell, xenograft models, and so on.

**Results:**

miR-210 is significantly expressed in PDAC exosomes and malignant cells. High miR-210 significantly facilitated tumor angiogenesis, cell invasion, and proliferation in PDAC cells. Further mechanistic detection revealed that miR-210 negatively regulated EFNA3 expression and participated in the PI3K/AKT/VEGFA or Wnt/Β-catenin/RHOA pathways, thus promoting tumor angiogenesis and cellular permeability. PDAC cells promote endothelial angiogenesis or permeability via miR-210 transmission by tumor exosomes. Exosomal miR-210 promotes PDAC progression *in vivo*. Further detection of PDAC plasma exosomal miR-210 suggests that exosomal miR-210 expression was high and significantly associated with vascular invasion and TNM stage and was an independent risk factor for PDAC overall survival.

**Conclusions:**

PDAC cell-secreted exosomes could promote angiogenesis and cellular permeability of neighboring endothelial angiogenesis or permeability via miR-210 transmission. Exosomal miR-210 may play important roles in tumor biology and may be a useful prognostic marker in PDAC.

## 1. Background

Pancreatic ductal adenocarcinoma (PDAC) is currently one of the most malignant and lethal tumors worldwide [1]. In recent decades, PDAC has been the only tumor with an increasing mortality rate, despite marked improvements in prognosis with various other cancers due to tremendous advances in diagnosis and treatment techniques [2]. According to the latest report, the median survival time of PDAC patients is approximately 6 months and the 5-year survival rate is less than 8% [3]. Radical resection is still the most important treatment at present. However, nearly 80% of PDAC patients have been in the middle or advanced stage and have lost their opportunity for surgery at the first admission and less than 20% of PDAC patients have the opportunity for radical surgery [4]. Even so, the 5-year survival rate after surgery is only approximately 25% [5]. Therefore, the key to the treatment of PDAC is to improve the early diagnosis rate and to find new therapeutic targets that inhibit invasion and metastasis.

Exosomes are cell-secreted, nanosized bilayer membrane extracellular vesicles with diameters ranging from 40–100 nm [6]. Exosomes can carry a different number of bioactive molecules, including proteins, DNA, various types of RNAs (such as miRNAs, mRNAs, lncRNAs, and circular RNAs), lipids, and metabolites according to the cells of origin via cellular communication [7–9]. Exosomes were initially viewed as waste products produced during different physiologic cell metabolisms [10]. However, increasing evidence has shown that exosomes play an important role in cellular communication by transferring exosomal molecules to recipient cells, thus resulting in a series of physiological changes, including promoting cell growth, proliferation, and migration [11, 12]. Exosomes can be secreted by various cells, including tumor cells, and are commonly found in various human body fluids, including plasma and ascites [13]. In turn, various abnormal biological alterations of different cells (including tumor cells) can also be reflected by detecting the expression of exosomal molecules (including miRNAs) in body fluids [14]. Recent studies have shown that tumor cells can secrete many more exosomes than normal cells, and abnormal exosomes can be easily detected in various body fluids, such as urine, saliva, ascites, or blood [9]. Many studies have shown that the abnormal expression of miRNA in tumor exosomes is closely related to various biological changes, such as angiogenesis, tumor invasion, and metastasis [14, 15]. The detection of abnormally expressed molecules in exosomes may play an important role in the early diagnosis, prognosis, or monitoring of the treatment of cancer and provide new therapeutic targets to treat tumors via exosomes. Therefore, the detection of exosome-based molecules in body fluids may provide novel methods for the early diagnosis and treatment of PDAC.

In this study, we identified miR-210 in PDAC exosomes by miRNA microarray analysis, and the biological functions and detailed regulatory mechanisms were also detected. We also visualized miR-210 transmission between PDAC cells and endothelial cells. Finally, the expression of miR-210 in plasma exosomes was further detected and its relationship with clinical survival was analyzed.

## 2. Methods

### 2.1. Cell Culture and Transfection

The cell lines involved in the study were authenticated by STR profiling, and Hs766T and HUVECs were purchased from American Type Culture Collection (ATCC, Manassas, USA). Other PDAC cell lines, including Hs766T-L1, Hs766T-L2, and Hs766T-L3, are first-, second-, and third-generation primary cells from nude mouse liver metastatic tissue of Hs766T, respectively, as described in our previous paper [16]. Cells were incubated in 5% CO_2_ at 37°C and cultivated in RPMI 1640 (Gibco, USA) or DMEM (Gibco, USA) supplemented with 10% FBS (HyClone, USA). Transfections of plasmid and shRNA were performed using the transfection reagent Lipo 3000 (Invitrogen, USA) according to the manufacturer's instructions.

### 2.2. Cell Invasion Experiment

Cell invasion was examined using Transwell assays. In brief, control or transfected PDAC cells resuspended in a FBS-free medium were cultured in Transwell migration chambers (8 *μ*m polycarbonate filter) precoated with Matrigel (BD, USA). After 36 h, cells that failed to invade from the top of the membrane were removed with a cotton swab, and cells on the bottom of the membrane were fixed and then stained. Invaded cell images from five randomly selected grid squares were counted.

### 2.3. EdU Incorporation Assay

The EdU assay was conducted with a keyFluor488 Click-iT EdU Detection Kit (KGA331-100, KeyGene, Nanjing, China) according to the manufacturer's instructions. In brief, control or transfected PDAC cells were incubated with EdU, fixed and permeabilized, and then incubated with Click-iT. After further incubation of the nucleus with DAPI (Beyotime, China), the slides were finally imaged with a fluorescence microscope.

### 2.4. RNA Isolation and PCR Analysis

Total RNA from PDAC cells or clinical samples was extracted by using TRIzol and TRIzol LS (Thermo, USA) reagent according to the manufacturer's instructions. The cDNA was generated with PrimeScript RT Reagent Kit, with gDNA Eraser (TaKaRa, Japan), or Mir-X miRNA qRT-PCR SYBR Kit (Clontech, Japan) according to the manufacturers' protocols. Real-time PCR was performed using SYBR Premix Ex Taq (TaKaRa, Japan) on a CFX96 Real-Time System (Bio-Rad, USA) based on the instructions.

### 2.5. Luciferase Reporter Assays

Different treated cells were transfected with pGL3 EFNA3 or pGL3 mut-EFNA3 plasmid (Sangon Biotech, China) and the internal control pRL-TK Renilla Luciferase Plasmid (Promega, USA) combined with miR-210 mimics in 96-well plates. After incubation, the cells in the plate were harvested and processed according to the manufacturer's protocol (Dual-Luciferase Reporter Assay System, E1910, USA). The final firefly luciferase activity was normalized to Renilla luciferase activity.

### 2.6. Western Blot Analysis

WB analysis was performed as described previously [17]. Generally, total protein was extracted by RIPA Lysis Buffer (Thermo, USA). Protein was measured, separated, and transferred to PVDF membranes (Millipore, USA) after further incubation with primary and secondary antibodies overnight at 4°C. The final protein was visualized by the Super ECL Detection Kit (KeyGEN BioTECH, China). The antibodies used in this study were as follows: anti-HIF-1*α* (1 : 1000, #36169S, Cell Signaling, USA), anti-EFNA3 (1 : 1000, ab153706, Abcam, USA), anti-AKT (1 : 1000, 4691, Cell Signaling, USA), anti-p-AKT (1 : 1000, 4060, Cell Signaling, USA), anti-VEGFA (1 : 1000, #65373, Cell Signaling, USA), anti-Wnt3a (1 : 1000, #2721, Cell Signaling, USA), anti-*β*-catenin (1 : 1000, #9562, Cell Signaling, USA), anti-RhoA (1 : 600, #2117, Cell Signaling, USA), and anti-*β*-actin (1 : 5000, 20536-1-AP, Proteintech, USA).

### 2.7. Exosome Experiments

As described previously, PDAC cells were cultured with exosome-depleted FBS, and 10 ml of conditioned medium was used for exosomes extraction with a Total Exosome Isolation Kit (Thermo, USA) according to the manufacturer's protocol. The FLUOROCET Ultrasensitive Exosome Quantitation Assay Kit (SBI, USA) was used to ensure the same number of exosomes was used in each experiment. As discussed before, 5 × 108 exosomes were used for exosomal RNA extraction and 1 × 10^8^ exosomes were used for exosome-stimulation experiment *in vitro*. The size and morphology were further confirmed by transmission electron microscopy (TEM) and exosomal marker tests, please refer to the previous studies [16–18].

### 2.8. PDAC Patients and Clinical Samples

Seventy-three blood samples were obtained from patients who underwent pancreaticoduodenectomy surgery at Southwest Hospital, the Affiliated Hospital of North Sichuan Medical College, or the Central Theater Command General Hospital of PLA from 2014 to 2018. This study was approved by the Ethics Committee of Southwest Hospital and the Affiliated Hospital of North Sichuan Medical College, and all patients provided written informed consent. The clinical and pathological characteristics of PDAC patients are summarized in Table 1. Formal informed consent was signed by all patients before collecting blood samples.

### 2.9. Tube Formation Assay

The formation of capillary-like structures was assessed in a 24-well plate using growth factor-reduced Matrigel (BD, CA, USA). HUVECs (4 × 10^4^ cells) were plated on top of Matrigel after transfection. After 24 h, the cells were visualized under a contrast phase microscope. The total tube area was quantified as the mean pixel density obtained from image analysis of three random microscopic fields using ImageJ software.

### 2.10. Animal Experiment

Animal experiments in this study were approved by the Institutional Animal Care and Use Committee of Southwest Hospital, Chongqing, China. As previously described, 4- to 6-week-old male nude mice were used and anesthetized with 1% sodium pentobarbital. Then, a median abdominal incision was made to expose the spleen and pancreas, and 5 × 10^6^ PDAC cells (in 100 *μ*l of PBS) were injected to the head of the pancreas. After replacing the pancreas and closing the abdomen with a continuous 4-0 braided silk suture, the mice were imaged by the IVIS Lumina II System (Caliper Life Science, USA) each week until the experiments were completed.

### 2.11. Statistical Analysis

All analyses were performed using SPSS 26.0 software (IBM, USA), and the graphs and diagrams were generated by GraphPad Prism 9 (LLC, USA). Experiments were performed in triplicates. *χ*^2^ test was used to compare the correlation between clinical information and miR-210 expression. Student's *t* test or one-way analysis of variance (ANOVA) was used to analyze statistics between two groups or multiple groups, and a *P* value <0.05 was considered to be statistically significant.

## 3. Results

### 3.1. Tumor-Derived Exosomes Promote Tumor Angiogenesis in Recipient PDAC Cells

**Figure 1:**
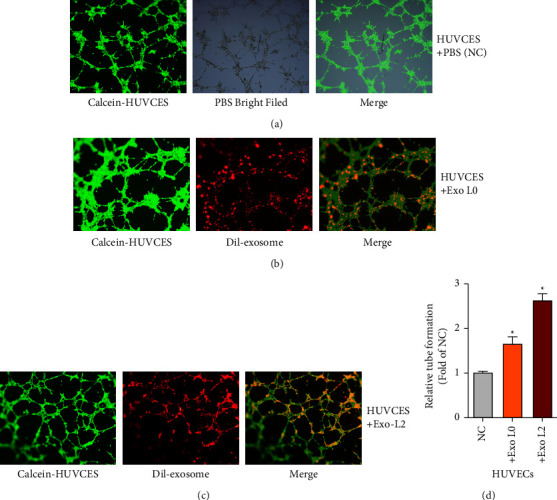
Tumor-derived exosomes promote tumor angiogenesis in recipient PDAC cells The *in vitro* angiogenesis abilities of the indicated treated HUVECs (A–D) were measured by tube formation assays. Magnification, ×200.

To clarify whether tumor exosomes have a stimulatory influence on endothelial cells, three PDAC cell lines were used, and the invasive or metastatic ability of the three PDAC cell lines from high to low was Hs766T-L3, Hs766T-L2, and Hs766T (i.e., Hs 766T-L0), respectively [16]. HUVECs with PBS or exosomes, which were marked with Dil dye, were cocultured from Hs766T or Hs766T-L2 cells and found that exosomes from Hs766T-L2 or Hs766T cells significantly increased the angiogenesis of HUVECs, as many more tube-like structures were significantly investigated compared to the PBS group (Figures 1(a)–1(d)).

### 3.2. Identification of miR-210 in PDAC Exosomes

**Figure 2:**
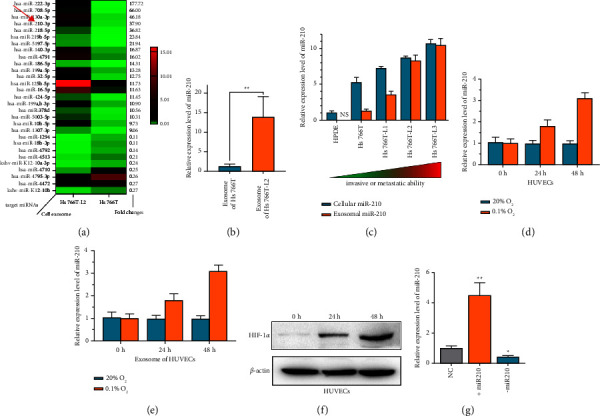
The identification of miR-210 in PDAC exosomes. (a) Heatmap of the screened miRNAs of exosomes from Hs766T and Hs766T-L2 cells. The fold change in each screened miRNA is attached in the right column. (b) Relative miR-210 expression in exosomes of Hs766T and Hs766T-L2 cells was measured by qRT-PCR. (c) Relative miR-210 expression in the indicated PDAC or normal cell lines was measured by qRT-PCR. (d) Relative miR-210 expression in the indicated HUVECs was measured by qRT-PCR. (e) Relative miR-210 expression in the indicated exosomes of HUVECs was measured by qRT-PCR. (f) Relative HIF-1*α* expression in the indicated HUVECs was measured by WB analysis. (g) Relative expression of miR-210 in the indicated treated cells was measured by qRT-PCR. +miR-210, miR-210 was overexpressed by miR-210 lentivirus; −miR-210, miR-210 was inhibited by miR-210 shRNA.

Many miRNAs in exosomes were found to play variable important roles in tumor biology [15, 19]. First, exosomal miRNA expression in the PDAC cell lines Hs766T and Hs766T-L2 to screen for possible useful tumor-related miRNAs were profiled. The top 30 differentially expressed miRNAs are shown in Figure 2(a). Of these differentially expressed miRNAs, it was noted that miR-210-3p (miR-210) was elevated in tumor exosomes, with a fold-change of 37.9 (Figure 2(a)). Then, the high expression levels of miR-210 were further confirmed by qRT-PCR in exosomes from Hs766T-L2 cells (Figure 2(b)). The cellular and exosomal miR-210 expression in Hs766T (the parent cell), Hs766T-L1, Hs766T-L2, and Hs766T-L3 (the daughter cell) cells was further detected. As the malignancy of PDAC cells increased, the expression level of miR-210 was also increased successively in both PDAC cells and their exosomes (Figure 2(c)). As previous studies reported that miR-210 was associated with hypoxia [20, 21], then HUVECs in normal or hypoxic conditions (oxygen content: 0.1%) were cultured. The results showed that after 24 h or 48 h, cellular or exosomal miR-210 was significantly increased under hypoxic conditions (Figures 2(d) and 2(e)). HIF-1*α* expression was also increased under hypoxic conditions (Figure 2(f)). Thus, it was speculated that miR-210 may have an important function in PDAC. Then, its upregulation (miR-210 lentivirus) or downregulation (miR-210 shRNA) in HUVECs was further confirmed (Figure 2(g)). Together, we screened miR-210 in tumor exosomes and further confirmed that miR-210 was highly expressed in malignant PDAC cells.

### 3.3. Promotion of Tumor Angiogenesis, Cell Invasion, and Proliferation in HUVECs by miR-210

**Figure 3:**
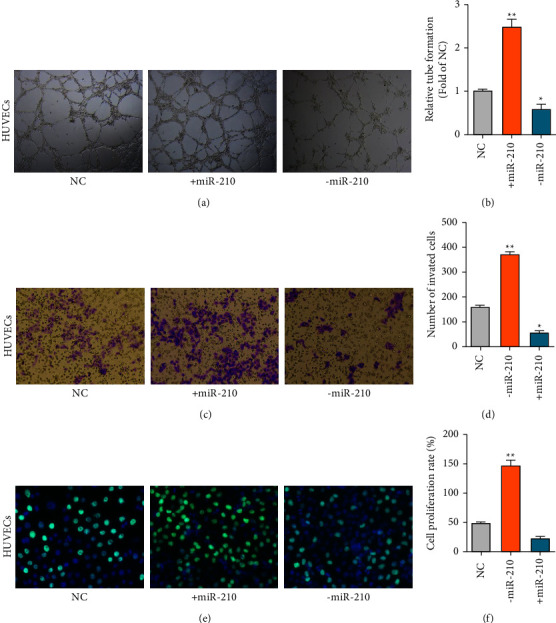
miR-210 promotes tumor angiogenesis, cell invasion, and proliferation in HUVECs. (a–b) The angiogenesis abilities of the indicated treated HUVECs measured by tube formation assays. Scale bars = 50 *μ*m. (c–d) The invasion abilities of HUVECs were measured by transwell assays. Scale bars = 50 *μ*m. (e–f) The proliferation abilities of the indicated treated HUVECs were measured by EdU assays. Magnification, ×200.

As miR-210 was highly expressed in malignant cells, we wondered whether miR-210 could promote angiogenesis, cell proliferation, or invasion. As expected, tube formation assays showed that miR-210 increased angiogenesis, while miR-210 shRNA decreased the angiogenesis of HUVECs (Figures 3(a) and 3(b)). Transwell invasion assays also revealed that miR-210 promoted cell invasion in HUVECs (Figures 3(c) and 3(d)). Furthermore, EdU assays showed that miR-210 overexpression increased cell proliferation, but the miR-210 inhibitor decreased cell proliferation in HUVECs (Figures 3(e) and 3(f)). Together, the results mentioned above suggest that miR-210 could promote angiogenesis, invasion, and proliferation.

### 3.4. Regulation of EFNA3 Expression by miR-210

**Figure 4:**
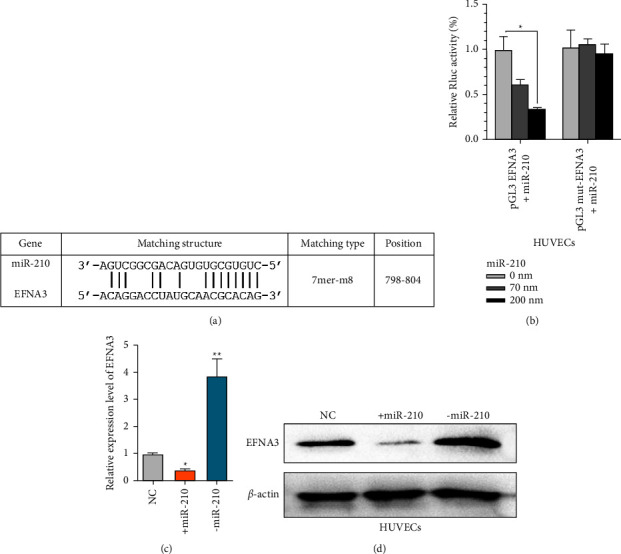
miR-210 regulates EFNA3 expression. (a) The prediction for miR-210 binding sites on the EFNA3 transcript. (b) Luciferase activity in HUVECs cotransfected with the indicated miR-210 concentration or EFNA3 luciferase reporter transcript. Data are shown as the ratio of firefly activity to Renilla luciferase activity. (c) Relative EFNA3 expression in the indicated HUVECs was measured by qRT-PCR. (d) Relative EFNA3 protein expression in the indicated HUVECs was measured by WB analysis.

To further determine the detailed molecular mechanism of miR-210, bioinformatics tools were used to analyze its possible molecular target gene. It was noted that EFNA3, a classic tumor suppressor, has 7mer-m8 molecular binding sites for miR-210 (Figure 4(a)), and previous studies have also reported that EFNA3 can be regulated by miR-210 in the nervous system [22]. Therefore, the regulation of EFNA3 by miR-210 was further verified. In the dual-luciferase reporter assays, wild-type or mutated EFNA3 3′UTR firefly luciferase plasmids, in which the miR-210 binding sites were deleted, were constructed. The results showed that cotransfection of wild-type pGL3 EFNA3 and miR-210 inhibited Rluc expression, and this inhibition was even more obvious in the 200 nM miR-210 group than in the 70 nM miR-210 group (Figure 4(b)). The qRT-PCR results showed that EFNA3 was significantly inhibited by miR-210 and was overexpressed when treated with miR-210 shRNA (Figure 4(c)). Furthermore, WB assays also suggested that EFNA3 protein was significantly reverse regulated by miR-210 transfection, which showed similar results (Figure 4(d)).

### 3.5. Inhibition of Tumor Angiogenesis, Cell Invasion, and Proliferation in PDAC Cells by EFNA3

**Figure 5:**
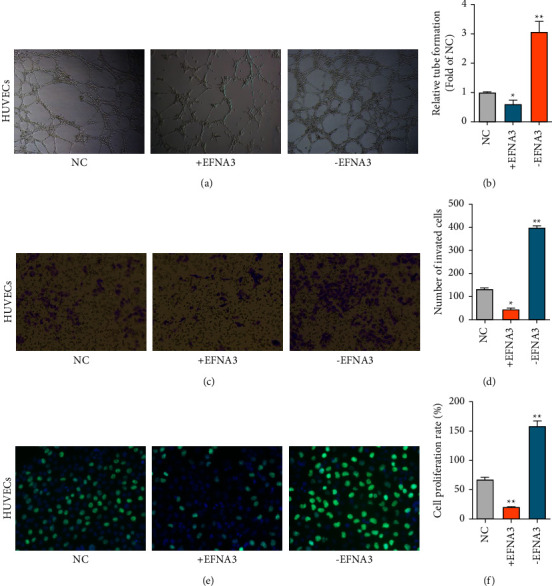
EFNA3 inhibited tumor angiogenesis, cell invasion, and proliferation in PDAC cells. (a–b) The angiogenic abilities of the indicated treated HUVECs measured by tube formation assays. Magnification, ×200. (c–d) The invasion abilities of HUVECs were measured by transwell assays. Magnification, ×200. (e–f) The proliferation abilities of the indicated treated HUVECs were measured by EdU assays. Magnification, ×200.

Then, the cellular functions of EFNA in HUVECs were studied. Tube formation assays showed that EFNA decreased angiogenesis, while EFNA shRNA increased angiogenesis in HUVECs (Figures 5(a) and 5(b)). Transwell assays also proved that EFNA inhibited cell invasion in HUVECs (Figures 5(c) and 5(d)). Furthermore, EdU assays proved that EFNA overexpression decreased cell proliferation, while the miR-210 inhibitor increased cell proliferation in HUVECs (Figures 5(e) and 5(f)).

### 3.6. Participation of miR-210 and EFNA3 in the PI3K/AKT/VEGFA and Wnt/Β-Catenin/RHOA Pathways

**Figure 6:**
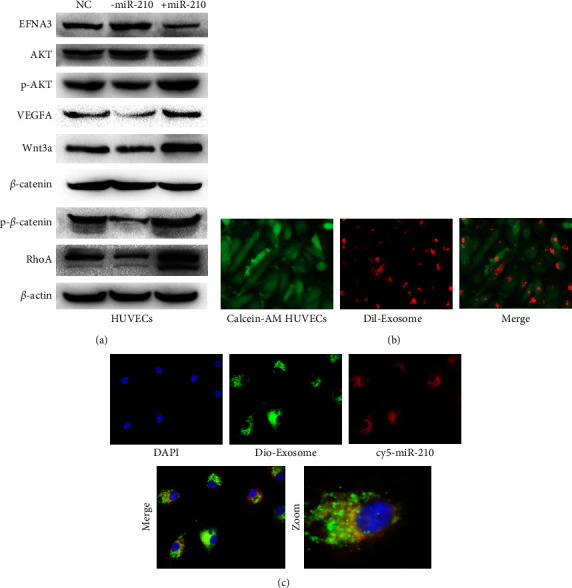
miR-210 or EFNA3 participates in the PI3K/AKT/VEGFA or Wnt/Β-catenin/RHOA pathways. (a) Relative indicated protein expression in the indicated HUVECs was measured by WB analysis. (b) Dil-labeled exosomes were added to Calcein AM-labeled HUVECs for cocultivation. Images were captured with a fluorescence microscope, and the white arrows indicate exosomes. Magnification, ×100. (c) Dio-labeled exosomes of ov cy5-miR-210 Hs766T were added to HUVECs for cocultivation. Images were captured with a fluorescence microscope. The green arrow indicates exosomes, and the pink arrow indicates cy5-miR signals. Magnification, ×200.

Then, the possible molecular pathways by which miR-210 or EFNA3 may be involved were studied. WB assays revealed that when miR-210 was overexpressed, the expression of EFNA3 was decreased, whereas the expression of p-AKT and VEFGA was obviously increased; it was also noticed that Wnt3a, catenin, and RhoA were also elevated (Figure 6(a)), which implies that miR-210 or EFNA3 participate in the PI3K/AKT/VEGFA or Wnt/Β-catenin/RHOA pathways.

### 3.7. Imaging Exosomal miR-210 Communication between PDAC and Endothelial Cells

Then, exosome communication between PDAC cells and endothelial cells was further explored. First, vascular endothelial cells were labeled with Calcein-AM*in vivo*, and then Dil-labeled PDAC exosomes were added to the culture medium of PDAC cells for coculturing. Finally, dotted, red PDAC exosomal signals in the cytoplasm of HUVECs were found, as shown in Figure 6(b). To further confirm the miR-210 communication between PDAC and HUVECs via exosomes *in vivo*, HUVECs with the red fluorescence signal (-cy5)-tagged miR-210 were first transfected. After two days of culture, the exosomes from the conditioned culture medium were extracted and labeled with a green fluorescence signal (-dio) for further cocultivation with HUVECs for 24 h. Interestingly, it was found that the red fluorescence signal (from miR-210) and green dotted-fluorescence signal (from exosome) were obviously mixed in the cytoplasm of HUVECs (Figure 6(c)). Together, the data mentioned above confirm the transmission of miR-210 between PDAC cells and HUVECs via exosomes.

### 3.8. Promotion of Tumor Progression via EFNA3 PI3K/AKT/VEGFA or EFNA3/Wnt/Β-Catenin/RHOA by Exosomal miR-210

**Figure 7:**
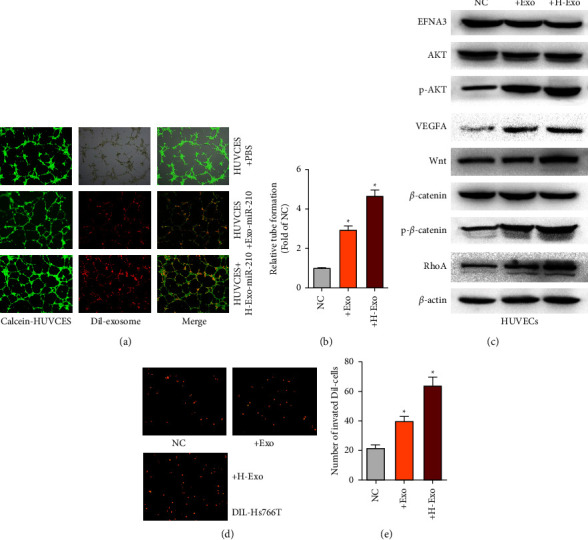
Exosomal miR-210 promotes tumor progression via EFNA3 PI3K/AKT/VEGFA or EFNA3/Wnt/Β-catenin/RHOA (a) The *in vitro* angiogenesis abilities of the indicated treated HUVECs were measured by tube formation assays. Magnification, ×200. (b) Relative expression of the indicated proteins in the indicated HUVECs was measured by WB analysis. (c) In the transwell assay, the tumor cells that passed through the endothelial monolayer were imaged by fluorescence microscopy. Magnification, ×100.

Then, whether exosomal miR-210 was biofunctional in PDAC was investigated. As shown in Figures 7(a) and 7(b), tube formation assays showed that exosomes with miR-210 overexpression or exosomes with miR-210 overexpression under hypoxic conditions significantly increased angiogenesis. Furthermore, we also found that the expression levels of p-AKT, VEFGA or Wnt3a, Catenin, and RhoA were elevated in exosomes with miR-210 overexpression or exosomes with miR-210 overexpression under hypoxic conditions, whereas EFNA3 expression was decreased (Figure 7(c)). Then, a cell permeability assay was performed. As shown in Figure 7(d), when exosomes overexpressing miR-210 or exosomes overexpressing miR-210 were added under hypoxic conditions, the number of invaded Hs766T cells was much greater than that in the NC group.

### 3.9. Promotion of Tumor Progression *In Vivo* by miR-210

**Figure 8:**
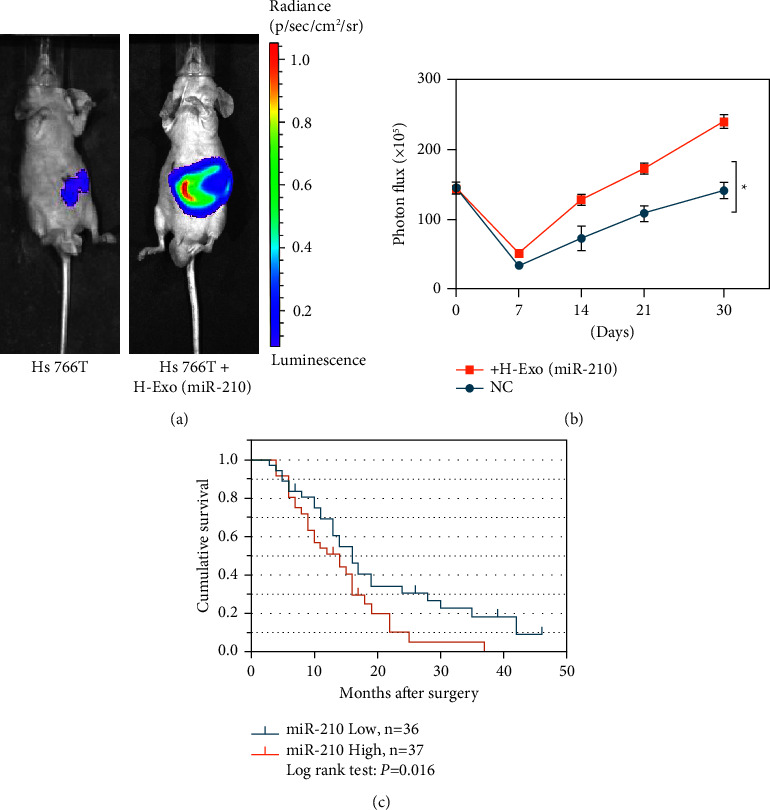
miR-210 promotes tumor progression *in vivo*. (a–b)In animal experiments, the luciferase intensities were measured each week after intrapancreatic injection with NC or the indicated treated cells. (c) K–M survival curves for the overall survival of 73 PDAC patients according to the relative expression of exosomal miR-210.

The results mentioned above suggest that exosomal miR-210 plays important roles in tumor progression *in vitro*. To further detect the roles of exosomal miR-210*in vivo*, an in situ PDAC model in nude mice was established. First, Hs766T cells with H-ExomiR-210 were cultured, and these cells were suspended in H-ExomiR-210 before the experiment. Then, 10^6^ cells were injected into the head of the pancreas of four- to six-weeks-old male nude mice. The luciferase intensity was assessed each week after injection to confirm tumorigenesis in situ (Figure 8(a)). The results showed that H-ExomiR-210 increased tumorigenesis, as intense luciferase signals compared to the NC mice, were detected.

### 3.10. Exosomal miR-210 Is an Independent Risk Factor for PDAC Patient Survival

Plasma samples from 73 clinical PDAC patients were further collected, and exosomal total RNA from plasma was extracted for further miR-210 detection. It was found that exosomal miR-210 was significantly associated with lymphatic invasion, vascular invasion, and TNM stage (Table 1). Subsequent survival analysis revealed that exosomal miR-210 and TNM stage were significantly related to poor survival rates in PDAC patients, and further multivariate analyses revealed that exosomal miR-210 expression was an independent risk factor for PDAC survival (Table 2).

## 4. Discussion

In this study, we screened miR-210 via miRNA microarray analysis of PDAC exosomes. Further research revealed that miR-210 regulates EFNA3 expression and that miR-210 or EFNA3 participate in the PI3K/AKT/VEGFA or Wnt/*β*-catenin/RHOA pathways. Also, miR-210 communication between PDAC cells and endothelial cells based on exosome transmission was proved. Finally, we also detected plasma exosomal miR-210 expression in clinical PDAC patients and found that high exosomal miR-210 expression is correlated with tumor progression and is an independent risk factor for PDAC survival.

There is no doubt that tumor angiogenesis is essential for tumor initiation, progression, and metastasis [23]. On the one hand, similar to nontumor tissues, tumor cells require angiogenesis to provide nutrients, oxygen, and metabolites; on the other hand, hematogenous tumor metastasis also depends on angiogenesis [24]. Tumors cannot grow without sufficient development of new blood vessels. Thus, targeting angiogenesis, especially endothelial cells, is one of the most important and common therapeutic targets in nearly all tumors. For example, bevacizumab, a humanized monoclonal antibody targeting VEGF-A, was used as monotherapy or in combination with other anticancer agents for various types of cancer, including HCC and colorectal cancer [25]. Nevertheless, the therapy seems not effective enough in PDAC, as clinical trials of gemcitabine plus antiangiogenic therapy with bevacizumab or axitinib failed to reach their primary endpoint of OS, and the possible reasons have been explored to date [26]. Studying angiogenesis has been an interesting topic in PDAC. On the one hand, PDAC is a hypovascular, hyperstroma, and poorly perfused tumor, and its major pathological feature is high levels of fibrosis, called desmoplasia [27]. Desmoplasia in PDAC leads to vasculature collapse that hinders vessel formation and inhibits drug penetration and uptake. Thus, some studies suggest that angiogenesis is not active enough in PDAC [28]; on the other hand, PDAC is characterized by rapid growth, recurrence, or metastasis, which definitely rely on an abundance of nutrients, especially the glucose supply itself [28, 29]. This paradox has resulted in relatively few studies on PDAC angiogenesis. Recently, a study revealed long, “hairy”-like projections on the basal surface of microvessels in PDAC, which means vascularization in PDAC is characterized by a high microvascular density, impaired microvessel integrity, and poorly perfused vessels [30]. Consistently, our results showed that PDAC could promote both angiogenesis and vascular permeability via exosomes and miR-210. This suggests that PDAC may be characterized as a hypovascular tumor on a macroscopic view or also a hypervascular (aberrant vessels) tumor on a microscopic view. The massive deviant, high-permeability microvessels may be part of the reasons why PDAC progresses or metastasizes rapidly.

Tumor-derived exosomes (TEXs) have attracted increasing interest in different tumors, as exosomes have rich endogenous contents that reflect the pathophysiology status and genic information of tumor cells [31]. Large amounts of DNA and different kinds of RNAs or proteins were found to be related to tumor initiation, progression, or prognosis; thus, TEXs are increasingly being recognized as potential tumor biomarkers. Recently, abundant miRNAs of TEXs have emerged in various tumors. A growing number of miRNAs have been shown to regulate nearly all physiological processes of tumor biology, including initiation, invasion, and metastasis [32, 33]. In PDAC, many oncosuppressor miRNAs have been identified in recent studies; for example, low miR-200 family expression was found to promote EMT, cell proliferation, and apoptosis [34]. Other similar tumor inhibitor miRNAs include miR-192, miR-26, and miR-34a [35]. Oncogenic miRNAs in TEXs were mainly focused, as the RNA levels in TEXs are relatively scarce, which is difficult for both to detect. Unlike detecting miRNAs in frozen tissue samples translationally, detecting miRNAs in TEXs has obvious advantages compared to detecting miRNAs in frozen tissue. First, exosomes can protect miRNAs from RNA degradation, and studies have shown that miRNAs in plasma exosomes are quite stable under different storage conditions. When exosomes were stored at −20°C, the overall amount of plasma miRNA was barely impacted for at least 5 years. Second, miRNAs in TEXs are easy to obtain in clinical practice (via blood drawn) compared with tumor or metastatic samples. Third, miRNAs in TEX exosomes can be continuously detected both before and after surgery. Thus, studying miRNAs in TEXs may be a convenient but effective approach in clinical practice. In this study, miR-210 in PDAC TEXs was detected. It was also found that the high level of exosomal miR-210 in PDAC was correlated with the progression and prognosis of PDAC, which suggests that the detection of exosomal miRNA in blood can be an expedient and valid approach to monitor the biological behavior of tumors.

## 5. Conclusions

In this study, we identified oncogenic miR-210 in PDAC exosomes by miRNA microarray analysis. Then, it was confirmed that miR-210 could regulate EFNA3 expression; further mechanistic analysis revealed that miR-210 could promote tumor progression via the PI3K/AKT/VEGFA or Wnt/Β-catenin/RHOA pathways. miR-210 can be transported from PDAC cells to endothelial cells via exosome communication in living conditions. Finally, high exosomal miR-210 expression was found to be associated with PDAC invasion and prognosis. Therefore, high exosomal miR-210 may be an important clinical marker and may play important regulatory and diagnostic roles in PDAC.

## Figures and Tables

**Table 1:** Clinical characteristics and expressions of exosomal miR-222 in 73 pancreatic carcinoma patients.

Parameters	*miR-210*		
	High	Low	*P* value/*χ*^2^
All cases	36	37	
*Gender*			0.955
Female	7	7	0.003
Male	29	30	

*Age, years*			0.417
≤60	19	23	0.658
>60	17	14	

*Tumor location*			0.656
Head	27	31	0.862
Body or tail	9	6	

*Tumor size, cm*			0.050
≤2	7	15	3.857
>2	29	22	

*Neural invasion*			0.566
No	21	24	0.329
Yes	15	13	

*Duodenal invasion*			0.781
No	31	31	0.077
Yes	5	6	

*Differentiation*			0.148
Low	13	6	3.817
Median	20	27	
High	3	4	

*Lymphatic invasion*			**0.023**
No	18	28	5.161
Yes	18	9	

*Vascular invasion*			**0.013**
No	22	32	6.102
Yes	14	5	

*Liver metastasis*			0.092
No	28	34	2.840
Yes	8	3	

*TNM*			**0.047**
I or II A	15	24	3.946
IIB or III and IV	21	13	

**Table 2:** Univariate and multivariate survival analyses of the prognostic factors associated with survival in pancreatic carcinoma patients (*n* = 73).

OS	*Univariate analysis*			*Multivariate analyses*		
	Patients (*n*)	Median survival time	*P* value	HR	95% CI	*P* value
Gender, male/female	14/59	12/15	0.547			
Age, ≤60/>60	42/31	16/13	0.259			
Tumor location, head/body or tail	58/15	14/15	0.625			
Tumor size, ≤2/>2	23/50	17/14	0.077			
Duodenal invasion, yes/no	62/11	15/14	0.955			
Differentiation, low/median/high	19/47/7	16/14/42	0.089			
Lymphatic invasion, yes/no	46/27	16/14	0.069			
Vascular invasion, yes/no	54/19	16/9	0.075			
Liver metastasis, yes/no	62/11	16/14	0.152			
TNM, I, IIA/IIB, and III or IV	39/34	16/13	**0.022**			0.117
miR-222, high/low	36/37	16/12	**0.016**	1.885	1.009–3.232	**0.022**

## Data Availability

The data used in this study are available on request from the corresponding author.

## References

[1] Guo F., Cheng X., Jing B., Wu H., Jin X. (2022). FGD3 binds with HSF4 to suppress p65 expression and inhibit pancreatic cancer progression. *Oncogene*.

[2] Gupta A., Virnig B. A., Nipp R. D. (2022). Opioid Prescriptions and Survival in Pancreatic Cancer. *JCO Oncol Pract*.

[3] Siegel R. L., Miller K. D., Fuchs H. E., Jemal A. (2021). Cancer statistics. *CA: A Cancer Journal for Clinicians*.

[4] Sugumar K., Hurtado A., Naik I. (2021). Multimodal Therapy with or without Irreversible Electroporation for Unresectable Locally Advanced Pancreatic Adenocarcinoma: A Systematic Review and Meta-Analysis. *HPB (Oxford)*.

[5] Budigi B., Oliphant M., Itri J. (2021). Pancreatic Adenocarcinoma: Diagnostic Errors, Contributing Factors and Solutions. *Acad Radiol*.

[6] Azmi A. S., Bao B., Sarkar F. H. (2013). Exosomes in cancer development, metastasis, and drug resistance: a comprehensive review. *Cancer Metastasis Rev*.

[7] Vader P., Breakefield X. O., Wood M. J. (2014). Extracellular vesicles: emerging targets for cancer therapy. *Trends in Molecular Medicine*.

[8] Moore C., Kosgodage U., Lange S., Inal J. M. (2017). The emerging role of exosome and microvesicle- (EMV-) based cancer therapeutics and immunotherapy. *International Journal of Cancer*.

[9] Deng Y., Sun Z., Wang L., Wang M., Yang J., Li G. (2021). Biosensor-based assay of exosome biomarker for early diagnosis of cancer. *Frontiers of Medicine*.

[10] Qiu P., Zhou J., Zhang J., Dong Y., Liu Y. (2021). Exosome: the regulator of the Immune system in Sepsis. *Frontiers in Pharmacology*.

[11] Sun Z., Yang J., Li H. (2021). Progress in the research of nanomaterial-based exosome bioanalysis and exosome-based nanomaterials tumor therapy. *Biomaterials*.

[12] Chen Q., Li Y., Gao W., Chen L., Xu W., Zhu X. (2021). Exosome-mediated Crosstalk between tumor and tumor-associated Macrophages. *Frontiers in Molecular Biosciences*.

[13] Nasser M. I., Masood M., Adlat S. (2021). Mesenchymal stem cell-derived exosome microRNA as therapy for cardiac ischemic injury. *Biomedicine & Pharmacotherapy*.

[14] Padda J., Khalid K., Khedr A. (2021). Exosome-derived microRNA: Efficacy in cancer. *Cureus*.

[15] Li Z., Tao Y., Wang X. (2018). Tumor-secreted exosomal miR-222 promotes tumor progression via regulating P27 expression and Re-Localization in pancreatic cancer. *Cellular Physiology and Biochemistry*.

[16] Li Z., Jiang P., Li J. (2018). Tumor-derived exosomal lnc-Sox2ot promotes EMT and stemness by acting as a ceRNA in pancreatic ductal adenocarcinoma. *Oncogene*.

[17] Li Z., Yanfang W., Li J. (2018). Tumor-released exosomal circular RNA PDE8A promotes invasive growth via the miR-338/MACC1/MET pathway in pancreatic cancer. *Cancer letters*.

[18] Li J., Li Z., Jiang P. (2018). Circular RNA IARS (circ-IARS) secreted by pancreatic cancer cells and located within exosomes regulates endothelial monolayer permeability to promote tumor metastasis. *Journal of Experimental & Clinical Cancer Research*.

[19] Wang B., Mao J. H., Wang B. Y. (2020). Exosomal miR-1910-3p promotes proliferation, metastasis, and autophagy of breast cancer cells by targeting MTMR3 and activating the NF-*κ*B signaling pathway. *Cancer Letters*.

[20] Tan Z., Xu J., Zhang B., Shi S., Yu X., Liang C. (2020). Hypoxia: a barricade to conquer the pancreatic cancer. *Cellular and Molecular Life Sciences*.

[21] Wang X., Luo G., Zhang K. (2018). Hypoxic tumor-derived exosomal miR-301a Mediates M2 Macrophage Polarization via PTEN/PI3K*γ* to promote pancreatic cancer metastasis. *Cancer Research*.

[22] Hu Y. W., Jiang J. J., Yan G., Wang R. Y., Tu G. J. (2016). MicroRNA-210 promotes sensory axon regeneration of adult mice in vivo and in vitro. *Neuroscience Letters*.

[23] Sherwood L. M., Parris E. E., Folkman J. (1971). Tumor angiogenesis: therapeutic implications. *New England Journal of Medicine*.

[24] Lugano R., Ramachandran M., Dimberg A. (2020). Tumor angiogenesis: causes, consequences, challenges and opportunities. *Cellular and Molecular Life Sciences*.

[25] Mashreghi M., Azarpara H., Bazaz M. R. (2018). Angiogenesis biomarkers and their targeting ligands as potential targets for tumor angiogenesis. *Journal of Cellular Physiology*.

[26] Annese T., Tamma R., Ruggieri S., Ribatti D. (2019). Angiogenesis in pancreatic cancer: Pre-clinical and clinical studies. *Cancers*.

[27] Whatcott C. J., Diep C. H., Jiang P. (2015). Desmoplasia in primary tumors and metastatic Lesions of pancreatic cancer. *Clinical Cancer Research*.

[28] Komar G., Kauhanen S., Liukko K. (2009). Decreased blood flow with increased metabolic activity: a novel sign of pancreatic tumor aggressiveness. *Clinical Cancer Research*.

[29] Zhang Z., Ji S., Zhang B. (2018). Role of angiogenesis in pancreatic cancer biology and therapy. *Biomedicine & Pharmacotherapy*.

[30] Saiyin H., Ardito-Abraham C. M., Wu Y. (2015). Identification of novel vascular projections with cellular trafficking abilities on the microvasculature of pancreatic ductal adenocarcinoma. *The Journal of Pathology*.

[31] Luo R., Liu M., Yang Q. (2021). Emerging diagnostic potential of tumor-derived exosomes. *Journal of Cancer*.

[32] Gurtner A., Falcone E., Garibaldi F., Piaggio G. (2016). Dysregulation of microRNA biogenesis in cancer: the impact of mutant p53 on Drosha complex activity. *Journal of Experimental & Clinical Cancer Research*.

[33] Croston T. L., Lemons A. R., Beezhold D. H., Green B. J. (2018). MicroRNA regulation of Host Immune Responses following Fungal exposure. *Frontiers in Immunology*.

[34] Herreros-Villanueva M., Zhang J. S., Koenig A. (2013). SOX2 promotes dedifferentiation and imparts stem cell-like features to pancreatic cancer cells. *Oncogenesis*.

[35] Wald P., Liu X. S., Pettit C. (2017). Prognostic value of microRNA expression levels in pancreatic adenocarcinoma: a review of the literature. *Oncotarget*.

